# In sickness and in health: The functional role of extracellular vesicles in physiology and pathology in vivo: Part I: Health and Normal Physiology

**DOI:** 10.1002/jev2.12151

**Published:** 2022-01-18

**Authors:** Abi G. Yates, Ryan C. Pink, Uta Erdbrügger, Pia R‐M. Siljander, Elizabeth R. Dellar, Paschalia Pantazi, Naveed Akbar, William R. Cooke, Manu Vatish, Emmanuel Dias‐Neto, Daniel C. Anthony, Yvonne Couch

**Affiliations:** ^1^ Department of Pharmacology University of Oxford Oxford UK; ^2^ School of Biomedical Sciences Faculty of Medicine University of Queensland St Lucia Australia; ^3^ Department of Biological and Medical Sciences Faculty of Health and Life Sciences Oxford Brookes University Headington Campus Oxford UK; ^4^ Department of Medicine, Division of Nephrology University of Virginia Charlottesville Virginia USA; ^5^ Molecular and Integrative Biosciences Research Programme Faculty of Biological and Environmental Sciences University of Helsinki Helsinki Finland; ^6^ Division of Cardiovascular Medicine, Radcliffe Department of Medicine University of Oxford Oxford UK; ^7^ Nuffield Department of Women's and Reproductive Health University of Oxford Oxford UK; ^8^ Laboratory of Medical Genomics. A.C. Camargo Cancer Centre São Paulo Brazil; ^9^ Laboratory of Neurosciences (LIM‐27) Institute of Psychiatry São Paulo Medical School São Paulo Brazil; ^10^ Acute Stroke Programme ‐ Radcliffe Department of Medicine University of Oxford John Radcliffe Hospital, Headington Oxford UK

**Keywords:** exosomes, extracellular vesicles, in vivo, microvesicles, pathology, physiology

## Abstract

Previously thought to be nothing more than cellular debris, extracellular vesicles (EVs) are now known to mediate physiological and pathological functions throughout the body. We now understand more about their capacity to transfer nucleic acids and proteins between distant organs, the interaction of their surface proteins with target cells, and the role of vesicle‐bound lipids in health and disease. To date, most observations have been made in reductionist cell culture systems, or as snapshots from patient cohorts. The heterogenous population of vesicles produced in vivo likely act in concert to mediate both beneficial and detrimental effects. EVs play crucial roles in both the pathogenesis of diseases, from cancer to neurodegenerative disease, as well as in the maintenance of system and organ homeostasis. This two‐part review draws on the expertise of researchers working in the field of EV biology and aims to cover the functional role of EVs in physiology and pathology. Part I will outline the role of EVs in normal physiology.

## 1 INTRODUCTION

Extracellular vesicles (EVs) are membrane‐bound particles, and are classified according to their biogenesis. What were traditionally called exosomes are the smallest (c.30–150 nm); generated via the endolysosomal pathway, and are formed from the invagination of endosomes and stored in multivesicular bodies before release by exocytosis. Those which were called microvesicles (MVs), also known as microparticles or ectosomes, are larger EVs (c.100–1000 nm) and bud directly from the plasma membrane. Finally, apoptotic bodies are released after programmed cell death and remain the largest subcellular particle (c.1000–5000 nm). Whilst exosomes, MVs and apoptotic bodies are the three “traditional” subgroups of EVs, it is worth noting that additional, distinct subgroups such as apoptotic cell‐EVs (Caruso & Poon, 2018) and exomeres (Anand et al., 2021) are being increasingly recognised, emphasizing the heterogeneity of EVs (Scott et al., 2021; Zhang et al., 2018, 2019; Zijlstra & Di Vizio, 2018). There is considerable overlap in size distribution between some of the smaller EVs and thus the umbrella term “extracellular vesicle” has been adopted to cover all types of vesicles released by cells, with details on nomenclature being added post‐hoc. For example “small CD63+ EVs” being those expressing the exosome‐enriched tetraspanin CD63, and having a diameter of less than 200 nm (Beer & Wehman, 2017; Hessvik & Llorente, 2018; Ramirez et al., 2018; Théry et al., 2018). It should be noted that all of the size ranges quoted here are, much like the presence or absence of certain tetraspanins, subject to the way EV measurements are taken and how the EVs are isolated. Whilst there has been a significant increase in the number of EV publications in the last decade, the precise role of EVs in vivo remains far from certain. The main aim of this review is to provide an update on the excellent review of Yáñez‐Mó et al. (2015) on the role of EVs in physiology, with our focus here being to highlight our understanding of the functionality of EVs in vivo in different physiological systems. Our understanding of the biogenesis of EVs, along with selective packaging of cargo largely comes from in vitro studies and has been extensively reviewed elsewhere (Abels & Breakefield, 2016; Mir & Goettsch, 2020).

One of the principal reasons for the lack of progress in understanding the function of EVs in vivo is the fundamental technical challenges that confront investigators wishing to study them (Ramirez et al., 2018). Grouping EVs by size or density, even using a number of accepted techniques, will often generate results that can vary considerably between experiments and isolation procedures. MVs express many markers traditionally associated with exosomes (Kowal et al., 2016), and have been shown to be as small as 100 nm (Durcin et al., 2017; Kowal et al., 2016; Willms et al., 2018), yet the recognized range for exosomes lies anywhere between 30 and 150 nm (Willms et al., 2018), giving considerable scope for overlap between the two populations. Generally, the isolation of EVs according to size/subtype can be achieved by either size exclusion or antibody differentiation, but, as has been discussed elsewhere, both have intrinsic shortcomings (Ramirez et al., 2018; Théry et al., 2018; Veerman et al., 2021). These technical difficulties mean that few studies have isolated and characterised the in vivo functions of specific populations or speculated on the contributions of each population to the overall function of circulating EVs. To provide a comprehensive overview in this review, EV will be the predominant term used unless another specific nomenclature has been employed in the cited literature.

The aim of this review is to give the reader a “state‐of‐the‐art” overview of the field of in vivo EV research. Here, in part I, the role of EV signalling in normal physiology in different systems was explored.

## 2 MECHANISMS OF EV SIGNALLING

### 2.1 EV biogenesis

EVs are known to have a unique signature of lipids, proteins and nucleic acids, reflecting their cell and tissue of origin. Protein composition has been the most extensively studied; EVs are enriched for proteins in the tetraspanin family (CD63, CD9, CD81) (Jankovičová et al., 2020), which are thought to contribute to membrane remodelling to form the EV structure (Bari et al., 2011). However, as with much of the fundamental mechanistic EV research this has yet to be extensively studied in vivo. The composition of surface lipids has been less studied, although is thought to be key in the regulation of EV release, and in their final composition (Charoenviriyakul et al., 2018). EVs exhibit enrichment for a range of lipids including phosphatidylserine (PS), ganglioside, cholesterol, glycosphingolipids and ceramide (Chen et al., 2019). Interestingly, it has been shown that EVs can contain nucleic acid, of which mRNAs and miRNA have undergone the most study, which can be delivered to *and exert an effect* in the recipient cell (Valadi et al., 2007). Whilst investigation into the packaging and delivery of EV cargo has been extensively studied in cell culture systems, there is little knowledge of their relevance in vivo (O'brien et al., 2020). Several groups have made use of Cre‐Lox reporter systems to demonstrate cargo transfer to a range of tissues, although current evidence does not confirm whether mRNA or protein transfer is responsible for this effect (Ridder et al., 2014; Zomer et al., 2015). It is also unclear as to whether RNA packaging differs between EV subpopulations, with stoichiometric loading appearing to be heterogeneous (Chevillet et al., 2014; Wei et al., 2017), although evidence increasingly indicates that most extracellular RNA is extravesicular (Tosar et al., 2021). Intriguingly, pathologies that include some degree of inflammation, including ischemia, appear to significantly increase Cre‐reporter activation in mice, indicating that EV cargo delivery may be particularly important in some pathological or stress states (Ridder et al., 2014).

The biogenesis pathway has the capacity to influence the cargo sorted into the individual EV subtypes (Raposo and Stoorvogel, 2013). Exosome formation is largely regulated by the ESCRT (endosomal sorting complex required for transport) pathway and, as such, 0 ‘exosomes’ are enriched for associated proteins (Alix, TSG101, HSP), which are commonly used as EV‐associated markers (Théry et al., 2018), as well as endosome‐related proteins (Rab GTPase, SNAREs) (Raposo and Stoorvogel, 2013). However, ESCRT‐independent exosome release has also been reported, and is mediated by sphingomyelinase and ceramide (Dickens et al., 2017; Trajkovic et al., 2008). By contrast, MVs, deriving from blebbing, show greater enrichment for plasma membrane proteins. For example, the plasma membrane lipid phosphatidylserine is expressed at greater levels, but not exclusively, on MVs over exosomes (Matsumura et al., 2019; Wei et al., 2018). These differences have been exploited in attempts to isolate subpopulations of EVs to investigate any differences in functionality, or to use specific populations as biomarkers (Sharma et al., 2017). However, there are growing, valid concerns regarding the specificity of these defining markers, which is hindering effective research (Connor et al., 2010; Poon et al., 2019), especially in complex biological fluids.

EV biocomposition has also been shown to shift in response to stimulation, reflecting the physiological state of the cell/tissue of origin. Changes in the protein, lipid and nucleic acid profiles of EVs have consistently been reported, depending on the specific stimulus but many of these reports have been in controlled cell culture conditions. In vivo, where cascades of signalling pathways are often triggered by a basic stimulus such as endotoxin, it is unknown whether specific EV physicochemical properties are responsible for exerting their effect, or whether quantity is more important. In addition to our lack of understanding of the importance of composition versus numbers in vivo, we also lack understanding of the mechanisms driving the change in EV biocomposition. If these mechanisms are driven by disturbances in homeostasis, it is necessary to investigate them in an in vivo environment.

### 2.2 EV mechanisms of release

The exact mechanisms of EV release have been extensively reviewed elsewhere (Beer & Wehman, 2017; Hessvik & Llorente, 2018) but, in the broadest sense, they can be categorized into blebbing (for MVs from the cell surface) and exocytosis (for exosomes from the endosomal compartments). A considerable amount of in vivo evidence for the regulation of biogenesis and release comes from both *C.elegans* and *Drosophila* where knockdown and overexpression can be investigated easily (Beer & Wehman, 2017). Knockdown of many key proteins, such as the ESCRT machinery and Rab proteins, in mammals tends to cause embryonic lethality (Yu et al., 2014), making investigating their role in EV release in whole animals challenging. However, understanding organotropism, and the degree to which EVs have a specific ‘address’ or a specific function is only possible using whole animal systems.

Whilst it is clear that membrane dynamics and cell surface composition are key to EV biogenesis (Menck et al., 2017; Subra et al., 2007; Trajkovic et al., 2008), the role of different stimuli in generating an increase or decrease in EV release in vivo is yet to be determined. Moreover, the mechanisms governing the release of exosomes, versus those governing the release of MVs, are likely to differ. These differences may be key to the function of EVs in pathology. A number of pathologies, and homeostatic responses, display increased circulating EVs, which are thought to have a functional downstream role (Bei et al., 2017; Hazelton et al., 2018; Rackov et al., 2018). Whether this increase is a direct result of the disturbance, or is an attempt by the body to restore homeostasis, is yet to be determined. Moreover, it is unclear whether the mechanisms that govern basal production differ from those after insult. Once we understand more about how specific stimuli generate an increase in circulating EVs, we may be able to intervene in this process for therapeutic gain. Interestingly, similar cellular stimuli often elicit contradictory EV responses. For example, in their 2017 paper, Akbar and colleagues (Akbar et al., 2017) use two cytokines (IL‐1β and IL‐6), which converge on the same downstream signalling pathway (NF‐κB). Stimulation with these cytokines results in different EV responses; whilst IL‐1β increases levels of EVs released from endothelial cells, IL‐6 does not. Data such as these suggest that other factors, besides signalling cascades, instigate the production and release of EVs.

### 2.3 EV uptake and processing

The variable nature of EVs in terms of surface chemistry means that their signalling capacity is likely to be determined by their uptake mechanism and subsequent processing. EVs have been shown to interact with cells in a number of ways (Mulcahy et al., 2014), including phagocytosis (Chen et al., 2018; Feng et al., 2010), direct fusion with target cells via lipid rafts (Svensson et al., 2013) or via receptor interaction (Segura et al., 2005), presentation of biologically active molecules (Zhang et al., 2006), and uptake by endocytotic mechanisms (Mulcahy et al., 2014). A key question in basic EV biology is whether all cells take up all EVs by all mechanisms, or whether specific populations of EVs are preferentially taken up by specific mechanisms (Mulcahy et al., 2014). Indeed, the organotropism of particular EV populations is a topic under intense investigation (Gerwing et al., 2020; Hoshino et al., 2015; Mo et al., 2021). The adoptive transfer of non‐native EVs into naïve mice results in biodistribution which seems to be largely determined by route of injection, but can be influenced by cell source (Wiklander et al., 2015).

Despite our burgeoning knowledge of these mechanisms, there is still a fundamental chicken and egg debate regarding the specificity of the EV response. The donor cell may be specifically signalling to the recipient cell, and thus generating EVs with a unique ‘address’, or the donor cell may be generating EVs in a shotgun manner and the recipient cells are programmed to only take up specific subtypes. Currently we are too far from a good understanding of EV heterogeneity to accurately address this debate. By studying EV biology in vivo, we will form a more complete picture of the diverse roles of a mixed population of circulating vesicles in health and disease.

## 3 PLATELET BIOLOGY

Some of what we now consider to be the first evidence for the existence of ‘EVs’ (they were not referred to as such until relatively recently (Théry et al., 2018)) in vivo was in the field of coagulation. In 1946, Chargaff and West noted that platelet‐free plasma could clot on recalcification and that the clotting activity could be reduced by prior high‐speed centrifugation of 31,000 x g, suggesting that a sedimentable component within plasma was responsible for coagulation (Chargaff & West, 1946). Various studies went on to demonstrate that this component was to some degree responsible for the coagulative activity of plasma (Hougie, 1955; O'brien, 1955) but it was Peter Wolf who, in 1967, demonstrated that a phospholipid‐rich material derived from activated platelets was present in plasma and caused coagulation, describing it as “platelet dust” (Wolf, 1967). The vesicular material was dubbed as ‘Platelet factor 3′ or microparticles, and for a considerable period, platelet microparticles were thought to comprise 70–90% of all EVs present in the circulation. Only recently, data from cryo‐electron microscopy and improved (nano) flow cytometry of plasma EVs have revised this ratio to be closer to ∼30‐50% (Arraud et al., 2014; Berckmans et al., 2019). By their nature, the majority of what we know about platelet EVs is inherently in vivo as they can be studied in plasma, and most often from human subjects, provided that preanalytical factors are standardized. However, our understanding of their functional role is limited, as they are often only studied in in vitro assays.

Both resting and differentially activated platelets release a variety of EVs. In human studies, activating conditions such as cardiovascular stress (including exercise), hypoxia, inflammation and the consumption of a high fat diet all increase platelet EV levels in the circulation (Ayers et al., 2015). The diverse range of functional responses required from platelets, and the subsequent versatility of receptor‐mediated signalling, means that the subpopulations, morphology and the molecular content of EVs they produce is equally diverse (Aatonen et al., 2014; Brisson et al., 2017). Platelets are known to generate a range of different sized EVs from large to small, all with seemingly disparate functions. Large platelet EVs – often referred to as microparticles ‐ may contain mitochondria, in addition to their molecular cargo (Dean et al., 2009; Gasecka et al., 2019). Platelets from human plasma have also been shown to generate elongated tubular EVs (Brisson et al., 2017), which have also been shown in mice after arterial injury under flow conditions (Tersteeg et al., 2014). Smaller EVs (30 ‐150 nm) including those resembling exosomes have also been found in plasma (Heijnen et al., 1999). Previously, platelet EVs have been divided into four (Dean et al., 2009) to five (Pienimaeki‐Roemer et al., 2015) separate sub‐fractions from activated and senescent platelets, using gel filtration and density gradient ultracentrifugation, respectively, but modern techniques such as asymmetric field flow fractionation may change the number of the subpopulations (Multia et al., 2019). By electron microscopy, it is also possible to observe a number of morphologically variable and differentially originating subclasses (De Paoli et al., 2018), which may not be separable by the isolation techniques. These size‐separated fractions have both overlapping as well as distinct protein and lipid signatures (De Paoli et al., 2018).

As one of the older fields of EV biology, we know a significant amount about platelet EV contents, although in previous studies of isolated platelet EVs the rigor in their separation from the residual platelets has seldom been optimal. Platelet EVs contain proteins from the plasma membrane, cytosol, and organelles: adhesion receptors, coagulation and transcription factors, growth factors, active enzymes, cytokines and chemokines (Gasecka et al., 2019). GP IIa/IIIa (CD41/CD61), GP Ib (CD42b), P‐selectin (CD62P) and CD40L (CD154) are some of the key mediators of interaction with other cells in circulation and with matrices such as fibrin. Platelet EVs also carry proteins previously considered to be uniquely exosomal markers (CD9, CD63, CD81, HSP70, TSG101 (Ayers et al., 2015; Dean et al., 2009)). The cargo found within platelet EVs often provide a confusing picture of their function, as factors with opposing functions, e.g. pro‐ and anti‐coagulant (Tans et al., 1991) can be detected. They have also been shown to contain small metabolites (Puhka et al., 2017) and RNA (Laffont et al., 2013; Risitano et al., 2012), but the functional significance of these in vivo is largely unexplored. In addition to protein coding mRNA (Risitano et al., 2012), the platelet EV RNAome comprises microRNAs (miRNAs) (Xia et al., 2018), YRNAs (Driedonks et al., 2020) and circular RNAs (Preußer et al., 2018), originating from parent megakaryocytes. Autologous labelling experiments have shown that platelets circulate in human blood for about 10 days (Harker et al., 2000), but platelet EVs that are injected into peripheral blood of animals are cleared from circulation within 10–60 min (reviewed in (Ayers et al., 2015)) or in hours in humans (Rank et al., 2011). However, the degree to which this is reflective of clearance in normal physiology is unknown and challenging to explore in vivo. Interestingly, the timing and the removal mechanisms may depend on the EV subpopulation and characteristics, for example the presence or absence of phosphatidylserine (PS) on the surface of the EVs (Dasgupta et al., 2012). By using novel in vivo models such as the zebrafish (Verweij, Hyenne, et al., 2019), where the vasculature is easily visible and manipulable, we may learn more about the role of platelet EVs in normal physiology.

## 4 INFLAMMATION AND IMMUNITY

Cell‐to‐cell communication is an essential aspect of an effective immune system. It allows for the efficient transfer of information for a co‐ordinated response to protect the host from injury, infection and disease. Whilst soluble factors such as chemokines and cytokines are known modulators of the immune system, in recent years EVs have been identified as having a pivotal communicatory role in the initiation and resolution of inflammation (Buzas et al., 2014).

### 4.1 Immune system activation

**FIGURE 1:**
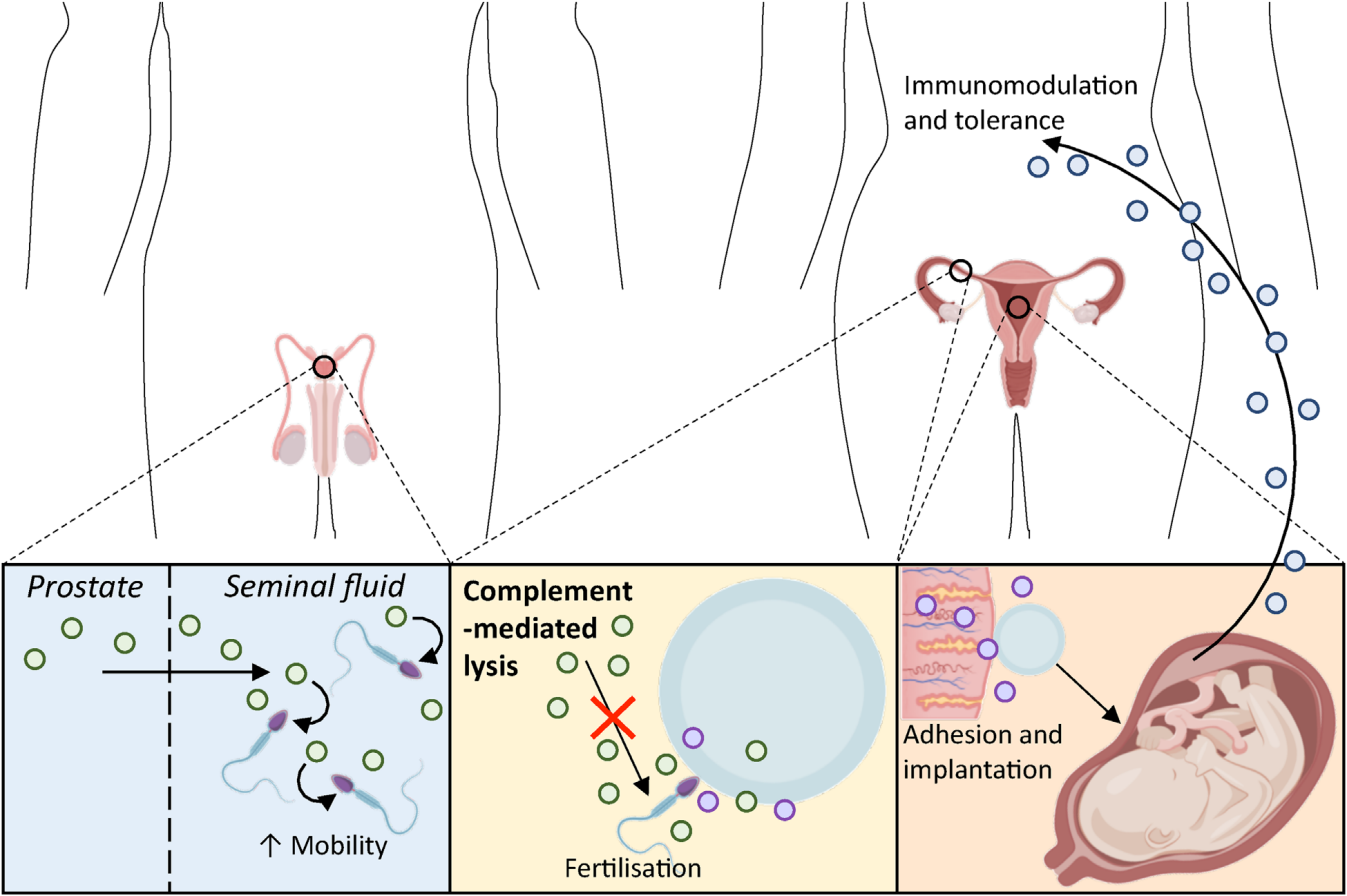
EV signalling has been associated with every stage of reproduction: from sperm motility and protection, fertilisation, adhesion and implantation, and well as maternal‐foetal signalling in later pregnancy to protect the foetus from the mother's immune system. Taken from refs: (Al‐Dossary et al., 2013; Carlsson et al., 2000; Greening et al., 2016; Hedlund et al., 2009; Kauma et al., 1999; Knight et al., 1998; Miyado et al., 2008; Nguyen et al., 2016; Orozco et al., 2009; Palmerini et al., 2003; Pap et al., 2008; Park et al., 2011; Ronquist, 2012; Rooney et al., 1993; Salomon et al., 2017; Schuh et al., 2004; Stenqvist et al., 2013; Tong & Chamley, 2015)

In response to injury or infection, the innate immune system is the first to respond and EV signalling has been shown to play a key role in this process (Hong, 2018; Zhou et al., 2020). EVs are released from neutrophils, monocytes and monocyte‐derived macrophages upon stimulation by inflammatory mediators, damage and/or pathogen associated molecular patterns (Headland et al., 2015, Timár et al., 2013). Certainly, an increase in circulating EVs, derived from these immune cells, has been detected in patients with a range of inflammatory and infectious diseases (Chen et al., 2020; Rossaint et al., 2016), which are thought to contribute to restoring homeostasis. EVs can target the insult directly; EVs derived from neutrophils have been shown to have anti‐microbial effects by inducing bacterial aggregation (Timár et al., 2013). Alternatively, EVs can enhance the immunological role of their parent cell. For example, neutrophil‐derived EVs increase expression of pro‐inflammatory molecules such as IL‐6 and adhesion molecules on endothelial cells (Kolonics et al., 2020), which facilitates their transmigration across endothelial barriers (Figure 1). These EVs are enriched for various chemokines, and so have been proposed to guide leukocytes to the site of inflammation (Lim et al., 2015; Nolan et al., 2008). Conversely, EVs from other sources, including cancer cells, can be taken up by leukocytes to trigger a response (Popēna et al., 2018), highlighting a bidirectional communicatory pathway. Most of our knowledge of EV signalling in the innate immune system has been acquired from in vitro studies; in vivo studies are lacking but necessary, given the interplay between the different components of the immune system and its interaction with different organs in the body.

Despite this lack of in vivo evidence, the role of EVs in the activation of immune cells remains one of the oldest areas in the field of EV biology, with what are now considered some of the most seminal papers. Raposo et al. (1996), in a revolutionary study, showed that exosomes released from B‐cell lines infected with the Epstein‐Barr Virus (EBV) were able to induce T‐cell proliferation and an antigen‐specific response. Protein analysis revealed that this could be attributed to EVs being enriched for Major Histocompatibility Complex (MHC) II and EBV‐specific proteins, suggesting the EVs were acting as antigen‐presenting vessels. Further characterisation in subsequent studies has shown EVs express both class I and class II MHC molecules, as well as adhesion and co‐stimulatory molecules. In this way, EVs are able to directly stimulate CD8+ and CD4+ T‐cells by binding to their relevant plasma membrane receptors (Nolte‐‘T Hoen et al., 2009). Early studies by Zitvogel et al. (1998) demonstrated that upon activation, DCs secreted antigen‐presenting exosomes, enriched for MHC complexes and T‐cell co‐stimulatory molecules, which elicited a T‐cell response to the antigen. They argued that this was a way of “priming the immune system”, promoting a specific cytotoxic response with higher immunogenicity. Thery et al.  (2002) went on to show that DCs pulsed with tumour peptides released immunogenic exosomes which induced a stronger CD8+ T‐cell anti‐tumour response than T‐cells incubated with the peptides alone, supporting the enhanced immunogenicity theory. This EV‐associated heightened inflammatory response has been exploited in trials of EV‐mediated anti‐tumour therapy. In vivo, it has been suggested that EVs display higher tumour antigen density and the presence of heat shock protein (HSP), which may act as an adjuvant in the activation of the immune system (Lv et al., 2012; Wolfers et al., 2001). As a result, it has been suggested that DCs can be primed with tumour‐associated proteins, inducing activation of leukocytes with tumour‐specific cytotoxicity as a vaccine‐related therapeutic, an approach which has now entered clinical trials (Escudier et al., 2005).

### 4.2 Immunosuppression and allergy

As well as activating the immune system, EVs are also capable of immune suppression, in a homeostatic capacity. For example, the cytotoxic activity of the NK‐derived exosomes was only evident on activated immune cells, but not resting cells (Lugini et al., 2012). This suppressive function of EVs is important during times where the pro‐inflammatory state may be detrimental, such as pregnancy. Placental exosomes and MVs are shed in large quantities during gestation (see *Part I*: *Reproductive Biology*) (Knight et al., 1998; Tong & Chamley, 2015) and have been associated with TNF‐family ligands FasL and TRAIL, driving apoptosis in activated lymphocytes (Kauma et al., 1999; Pap et al., 2008; Stenqvist et al., 2013). Indeed, FasL+ plasma EVs have been shown to induce CD4+ T‐cell apoptosis (Ren et al., 2011). Moreover, EVs derived from immature DCs have been shown to be enriched with TGF‐β1 and IL‐10, which inhibit T‐cell proliferation (Cai et al., 2012; Wan et al., 2018). In this way, EV‐mediated immunosuppression has an important role in preventing autoimmunity and chronic inflammation.

The concept of ‘tolerosomes’ was coined by Karlsson in the early 2000s, who demonstrated exosomal‐mediated immune suppression by intestinal epithelial cells (Karlsson et al., 2001), a function which may be key to the efficient development of an allergic response. This has also been demonstrated by Prado and colleagues (Prado et al., 2008), who isolated EVs from the bronchoalveolar lavage fluid of mice immunised against Olive Pollen Allergen, and adoptively transferred them into naïve mice. Exposure of these immunised mice to the allergen then resulted in a suppressed immune response and Th2 cytokine production. Similar results have been shown in response to ovalbumin (Hiltbrunner et al., 2016), with the authors suggesting that the mechanism is MHC and FasL mediated. This induction of immune tolerance has the potential to be exploited therapeutically for use in diseases such as graft‐vs‐host after transplant (Monguio‐Tortajada et al., 2014).

Within the context of the immune system, it is clear that EVs are capable of both activation and suppression of immune functions, and that this is likely to be context dependent. The interaction of immune cells with EVs, and each other, within the cascade of the inflammatory response means that studying this phenomenon effectively must happen, at least in part, in vivo.

## 5 THE CARDIOVASCULAR SYSTEM

In contrast to the plethora of studies investigating EVs in cardiovascular pathology, the study of the role of EVs in this system under healthy conditions is much less common, although some data suggest that EVs are involved in maintaining blood pressure (Good et al., 2020; Pironti et al., 2015). The vascular system maintains systemic blood pressure through the generation and release of vasoactive chemicals locally in the vessel wall. EVs can induce alterations in blood pressure through their enrichment with angiotensin II type I receptor (Pironti et al., 2015), but whether EVs in vivo alter the endogenous release of the primary vasodilator nitric oxide (NO) is unclear. A recent paper by Good et al. (2020) showed that circulating EVs isolated from normotensive rats and humans prevented acetyl choline‐induced vasodilation ex vivo, which the authors attributed to EV‐mediated inhibition of eNOS, suggesting EVs may indeed play a role in the regulation of NO production.

The study of EVs in the cardiovascular system in vivo has numerous difficulties. EVs are quickly cleared from the circulation and it may be difficult to measure transient changes in EV number, size and/or composition in vivo, and even more difficult to determine the destination of those injected systemically. Measuring brief changes, such as these, is complicated by limited blood volume in pre‐clinical models, and the ethical issues associated with rapid, repeated sampling in patients. Developing novel tools by crossing, for example, the fluorescent TIGER mice (Neckles et al., 2019) with endothelial specific CRE‐lines would allow for the functional study of endothelial cell‐derived EVs in vivo in real time. Similar use is being made of the zebrafish, a popular model for in vivo imaging due to its rapid life‐cycle and inherently manipulable genetic makeup (Hyenne et al., 2019; Verweij, Hyenne, et al., 2019). The vascular network of the zebrafish embryo is amenable to high speed temporal imaging (Hyenne et al., 2019; Verweij, Hyenne, et al., 2019) and therefore can be used to study EV basics such as rolling and adhesion, or organotropism. Indeed, Hyenne and colleagues effectively demonstrated the uptake of EVs in the caudal vein plexus of zebrafish, a region which has been likened to the vascular network of the liver (Hyenne et al., 2019). Here the haemodynamics are slower than in other blood vessels, allowing for the adhesion and arrest of EVs on the vascular wall, and their uptake in a dynamin‐dependent manner (Verweij, Revenu, et al., 2019). More recently, Scott et al. (2021) beautifully labelled endogenous cardiovascular EVs to image their distribution in the circulation, as well as their interaction between cell types in vivo. To recapitulate a more mammalian vascular network, it could be foreseen that high‐resolution in vivo imaging, such as that employed to image the living brain (Turcotte et al., 2019), could be combined with labelled EVs to enable us to determine whether these mechanisms are conserved between species.

## 6 THE RENAL SYSTEM

EVs are shed from various segments of the kidney directly into the urine, and so analysis of urinary EVs is a logical and novel approach to study the physiology and pathology in the kidney, and may even act as a surrogate for circulating EVs. As early as 2004, researchers performed a highly sensitive mass spectrometry analysis of urinary EVs (uEVs) enriched by ultracentrifugation from healthy urine (Pisitkun et al., 2004). Hundreds of proteins from nephron epithelial cells and the bladder were identified, opening up new discoveries for sensitive site‐specific, or disease‐specific, damage (bio‐) markers.

In vivo studies investigating the physiological role of EVs in the renal system under healthy conditions are very limited, indeed the majority of studies on EVs and the function of the kidney are found through studies in disease states (Hill et al., 2020; Jella et al., 2016; Munkonda et al., 2018). However, there is some suggestion that EVs contribute to the regulation of water and salt balance in healthy state. For example, Oosthuyzen et al., showed supportive evidence in an in vivo model that vasopressin regulates uptake of EVs in the collecting system (Oosthuyzen et al., 2016). Work in 2011 by Street et al. demonstrated that aquaporin‐2 (AQP2) is transferred via EVs to the collecting ducts after treatment with synthetic vasopressin (Street et al., 2011). Further to this, Miyazawa and colleagues have demonstrated that EVs enriched from AQP2 are found in human urine samples (Miyazawa et al., 2018). We know that AQP2 is an important regulator of water transport and therefore its functional transfer via EVs may be a novel mechanism in the retention of water within the kidney. As well as contributing to fluid balance, uEVs from healthy donors have been shown to be rich in antimicrobial proteins and peptides (Hiemstra et al., 2014). This proteomic data set suggests uEVs are innate immune effectors within the urinary tract.

There is also evolving evidence that EVs provide intra‐nephron communication along the urinary lumen, offering intra‐glomerular, glomerular‐tubular and intra‐tubular communication. However, as with other barriers, there is some discussion as to whether and how EVs might cross the glomerular filtration barrier. There is the potential for transcytosis through podocytes (Kerjaschki, 1990) or non‐vesicular EVs could be absorbed into the tubular system from the peritubular capillaries and secreted with EVs in urine. Again, in disease states the endothelial and podocyte barrier might be large enough to allow EVs (30 nm to 1000 nm) to transit (Ndisang, 2018) but this will be discussed in more detail in Part II.

Despite the availability of urine samples, the function of EVs in normal physiology of the renal system remains unclear and requires the translation of in vitro, scalable techniques into modifiable in vivo models. An excellent example of this is the study of tubular communication via EVs by Munkonda et al. (2018) and Gildea et al. (2014). These studies led to tubular interstitial communication being investigated in an animal model and the direct translation of these results into patients with IgA nephropathy (Lv et al., 2018). Clearly, such findings are often impossible to discover outside the context of the whole animal or patient. However, the field is increasingly moving towards the study of whole animal physiology using labelled EVs.

## 7 REPRODUCTIVE BIOLOGY

In addition to studying EVs in circulating cell populations, such as platelets and immune cells, the study of EVs in reproductive physiology is one of the older fields of EV research. Indeed “prostasomes” were first described before the term exosome was coined (Ronquist et al., 1978a, 1978b), and significant evidence for EV signalling at every stage of reproduction has been accumulated: from sperm and egg development, through fertilisation and implantation to maternal‐foetal signalling in later pregnancy (Simon et al., 2018) (Figure 1). EVs represent a key line of investigation in mechanistic research in normal gynaecological health and pre‐pregnancy signalling, during foetal development as well as during gynaecological pathology and diseases occurring during pregnancy (*see* Part II).

EVs were initially identified in seminal fluid in 1978 by Ronquist et al. (1978a). They have since been extensively characterised and defined as prostasomes, released from epithelial cells of the prostate gland into seminal fluid (Ronquist, 2012). These ∼150 nm diameter particles are produced by endosomal membrane invagination, creating a multivesicular body which fuses with cell membranes to release the prostasomes, a process comparable to exosome synthesis in other organ systems. Prostasomes have several key physiological roles in fertilisation. Prostasome fusion with sperm increases sperm motility, which is regulated by intracellular calcium [Ca^2+^] (Park et al., 2011). The mechanism for this was neatly demonstrated by Park et al.: prostasomes transfer CD38 into sperm, which induces cyclic ADP‐ribose (cADPR) production, with cADPR‐gated channels having a downstream role in motility. In addition to motility, other studies suggest prostasomes facilitate sperm‐egg fusion, which is necessary for fertilisation (Palmerini et al., 2003), that they protect sperm from complement‐mediated lysis by transferring an inhibitor of the membrane attack complex (Rooney et al., 1993) and have antibacterial activity (Carlsson et al., 2000).

In females, EVs have also been found in ovarian follicular fluid. Proteomic and genomic analysis of EVs from equine follicular fluid revealed 29 proteins not previously identified in exosomes, as well as 95 miRNAs (da Silveira et al., 2012). In the oviduct itself, EVs isolated from mouse luminal fluid transfer a calcium pump (PMCA4) into sperm (Al‐Dossary et al., 2013). PMCA4 knockout mice exhibit reduced sperm motility resulting in infertility, suggesting oviduct EVs signal between female genital tract and male gametes to facilitate fertilisation (Schuh et al., 2004). EVs have also been shown to facilitate sperm‐egg fusion in mice. Miyado et al. found that CD9 knockout mice are rendered infertile because there is impaired fusion of sperm and egg (Miyado et al., 2008). When incubating CD9‐/‐ sperm with CD9‐/‐ eggs, fusion could be recovered only by adding EVs derived from CD9+/+ eggs. In corroboration with this, the study also showed anti‐CD9 mAb inhibited the association of sperm with CD9‐containing vesicles, preventing sperm‐egg fusion. Finally, EVs have been shown to be released from endometrial epithelial cells, although evidence for their proposed function, mainly assisting adhesion and implantation, is derived only from cell culture experiments at this time (Greening et al., 2016; Nguyen et al., 2016). These experiments demonstrate that some of the older fields of EV research have progressed to studying the role of EVs in whole animals, and how important these experimental paradigms are to understand the field of EVs in physiology.

As well as pre‐pregnancy, fertilization and implantation, EVs have also been shown to play key roles during pregnancy. Specifically, research has demonstrated roles for EVs in early trophoblast development, maternal‐foetal signalling and in amniotic fluid signalling. Trophoblast EVs were first prepared in 1974, as an experimental model to interrogate the foeto‐maternal interface in vitro (Smith et al., 1974). It was not until some 20 years later that syncytiotrophoblast ‘vesicles’ were isolated from peripheral maternal plasma, demonstrating for the first time that these powerful packages might underlie signalling between foetus and peripheral maternal tissues in healthy pregnancy and disease (Knight et al., 1998), and further demonstrating the importance of studying such phenomena in the clinic. Extravillous trophoblast (EVT)‐EVs can be uniquely identified because they express HLA‐G and have been demonstrated to be present in the maternal circulation from the first trimester (Orozco et al., 2009). These data seem to suggest a role for EVs in maternal tolerance of, and adaptation to, pregnancy. One of the most intuitive functions for EVT‐EVs would be immunomodulation to ensure the uterus tolerates the foreign antigens presented by the developing foetus. Certainly, placenta‐derived EVs have been shown to reduce CD4+, CD8+ and NK cytotoxicity through Ig‐like receptors and the NK cell receptor NKG2D respectively (Hedlund et al., 2009; Pap et al., 2008). Limited in vitro experiments provide evidence to support this hypothesis, however in vivo work is still lacking.

During the later stages of pregnancy, EVs also facilitate communication between mother and foetus. The syncytiotrophoblast (STB) is the single cell layer between maternal and foetal blood. STB‐EVs are released directly into the maternal circulation and can be uniquely identified by the placenta‐specific protein: placental alkaline phosphatase (Knight et al., 1998). STB‐EVs have been found in increasing concentrations correlating with more advanced gestational age in peripheral plasma, consistent with increasing placental size (Salomon et al., 2017). However, the role of STB‐EVs is still under debate. They have been shown to carry numerous proteins including endoglin, plasminogen activator inhibitors, soluble fms‐like kinase (sFlt) and endothelial nitric oxide synthase (Guller et al., 2011; Motta‐Mejia et al., 2017), as well as microRNAs (Donker et al., 2012; Luo et al., 2009), tRNA (Cooke et al., 2019) and DNA (Gupta et al., 2004; Orozco et al., 2009). The physiological functions of these peripherally circulating EVs are broad and investigations have been a mix of in vitro and in vivo but their molecular contents and surface marker expression suggest the signalling power of peripherally circulating STB‐EVs should not be underestimated (Tannetta et al., 2017).

## 8 CENTRAL NERVOUS SYSTEM PHYSIOLOGY

The CNS remains one of the most challenging areas in which to study EV biology, largely due to the extremely heterogeneous nature of the tissue. The brain contains more different types of cells than any other organ in the body, and the cell populations are extremely dynamic and varied in their structures. This makes discovering cell‐specific markers a challenge and finding these rare markers in the circulation even more challenging. This particular problem has been abrogated recently by the advent of tissue dissociation techniques to isolate EVs directly from the brain (Vella et al., 2017). However, as a field, CNS EV research is still in its relative infancy compared to some of the other aspects of physiology.

**FIGURE 2:**
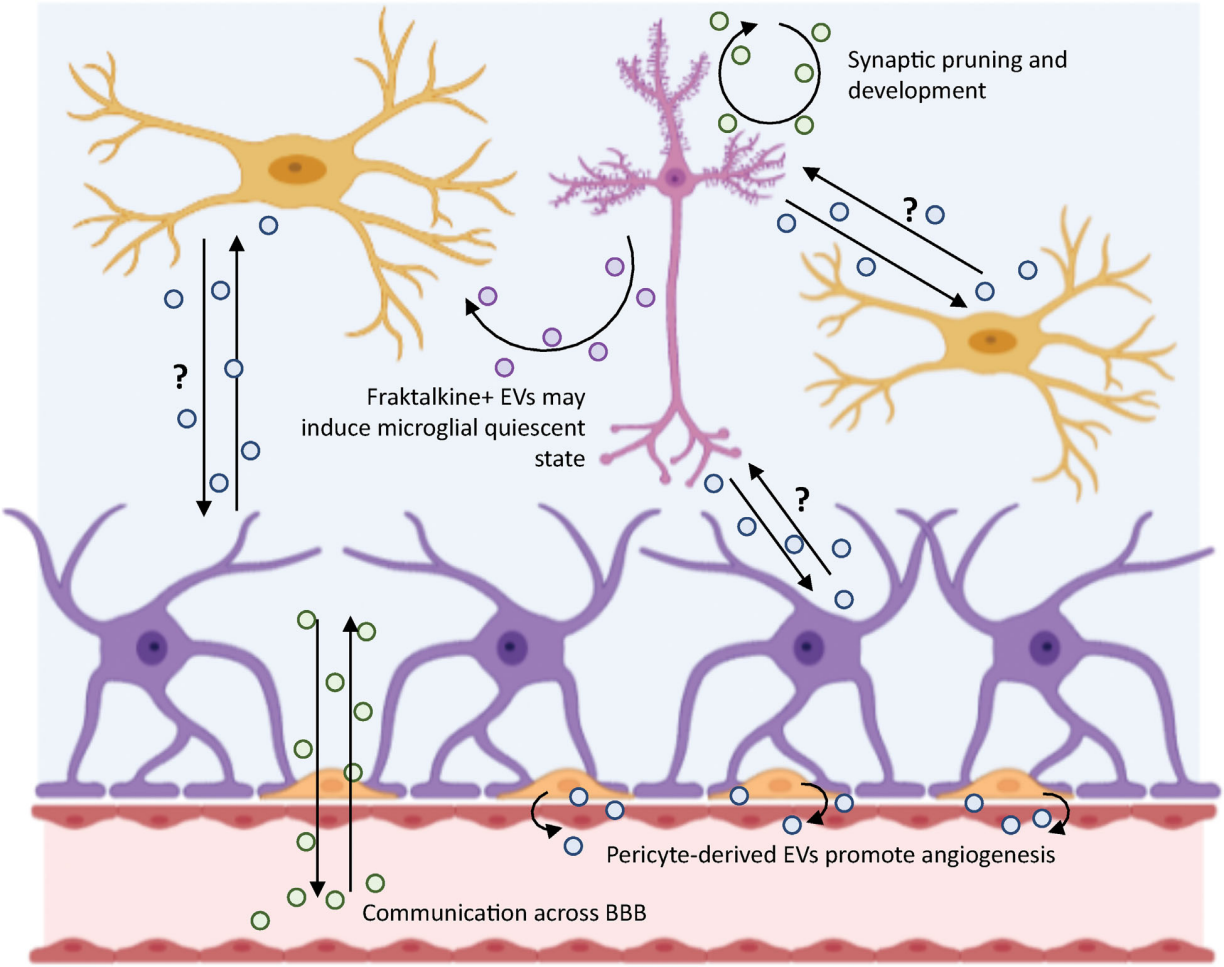
Examples of EV‐mediated signalling within the CNS, and between the CNS and the periphery under healthy conditions. EVs have been shown to be released from all cells of the CNS: neurons (pink), microglia (yellow), astrocytes (purple), pericytes (orange) and endothelial cells (red), as well as oligodendrocytes. However, many of the EV‐mediated signalling pathways between these cells remain speculative. Given the difficulties in studying CNS‐derived EVs in vivo, there remain many key questions about communication between the various cell types. The information presented here summarises what we know about communication between these cells in vivo and is taken from refs: (Alvarez‐Erviti et al., 2011; Brown et al., 2018; Dickens et al., 2017; Lachenal et al., 2011; Mayo & Bearden, 2015; Morales‐Prieto et al., 2018; Obermeier et al., 2013; Paolicelli & Ferretti, 2017; Paolicelli et al., 2014; Sokolowski et al., 2014; Tanaka et al., 2005; Vyas et al., 2014)

Within the CNS, EVs are released from all cell types, including neurons, astrocytes, microglia, oligodendrocytes and vascular cells (Figure 2). This neurovascular unit must act in concert for efficient and non‐pathological neurotransmission, a process which requires significant intercellular communication. Some of the first studies in this field demonstrated that increased neuronal activity is associated with increased EV release (Lachenal et al., 2011), a process thought to be vital for the effective maintenance of synapses. This neuronal activity‐dependent role of EVs is particularly important during development, when activity dependent‐pruning of synapses forms part of the normal development of the brain (Paolicelli & Ferretti, 2017). In addition to pruning, intercellular communication is key for the regional development of the CNS. For example, sonic hedgehog (Shh) signalling is known to regulate cortical development; Tanaka and colleagues have demonstrated that the flow of liquid around the developing mouse embryo releases EVs in a leftward direction (Tanaka et al., 2005). These EVs carry Shh and as such contribute to the lateralization of the CNS. Studies in chick embryos have also shown that Shh is found in EVs, and that different subpopulations of EVs (separated by size) express different molecules on their surface (Vyas et al., 2014). The authors suggest this results in regulated Shh signalling, by differentially expressing accessory molecules on the surface of different EV populations. These studies have been performed in vivo and provide crucial evidence that EVs are important during normal CNS development.

In the adult CNS, there are ongoing and essential intercellular interactions that need to be maintained in order for the CNS to function normally. Microglia need to remain in a quiescent, observant state. The blood brain barrier needs to remain intact. Astrocytes need to maintain healthy functionality. Direct evidence for the role of EVs in all of these processes is currently lacking, although data from other fields can be extrapolated. For example, the interaction of CX3CL1 (fraktalkine) on neurons, with its receptor CX3CR1 on microglia is known to induce a quiescent state (Paolicelli et al., 2014). The release of fraktalkine, sometimes referred to as the ‘find‐me’ signal, from apoptotic neurons is thought to be a mechanism of injury signalling to facilitate the clearance of neuronal debris after trauma (Sokolowski et al., 2014). In the immune system, EVs from endothelial cells have been shown to have fraktalkine on their surface, and to attract CX3CR1+ monocytes, acting as guidance cues (Brown et al., 2018). It is possible similar mechanisms exist in the CNS, but this is yet to be proven effectively.

The blood brain barrier (BBB) is made up of a unit of cells including pericytes, astrocytes and endothelial cells. The endothelial cells connect via tight junctions, preventing the normal migration of cells and large molecules seen in the fenestrated vasculature of the peripheral circulation (Obermeier et al., 2013). Early in vivo studies investigating the potential of EVs as a transport system for drugs demonstrated their capacity to cross the BBB (Alvarez‐Erviti et al., 2011). More recently, studies have shown that EVs pass from the periphery to the brain (Morales‐Prieto et al., 2018) and from the brain to the periphery (Dickens et al., 2017), making them an effective means of communication across an intact BBB. Whilst this has the potential to be exploited for therapeutic purposes, there is a considerable amount of intercellular communication between the cells of the neurovascular unit, and the role of EVs at this juncture should also be considered. For example, brain pericytes have been shown to produce EVs which are pro‐angiogenic, suggesting a role for EVs in regulation of normal growth and function at the BBB (Mayo & Bearden, 2015). However, as with the mechanisms of EV release, the complexity of this micro‐organ requires reductionist approaches and the majority of mechanistic studies of EVs at the BBB have been performed in cell culture.

## 9 MUSCULOSKELETAL PHYSIOLOGY

**FIGURE 3:**
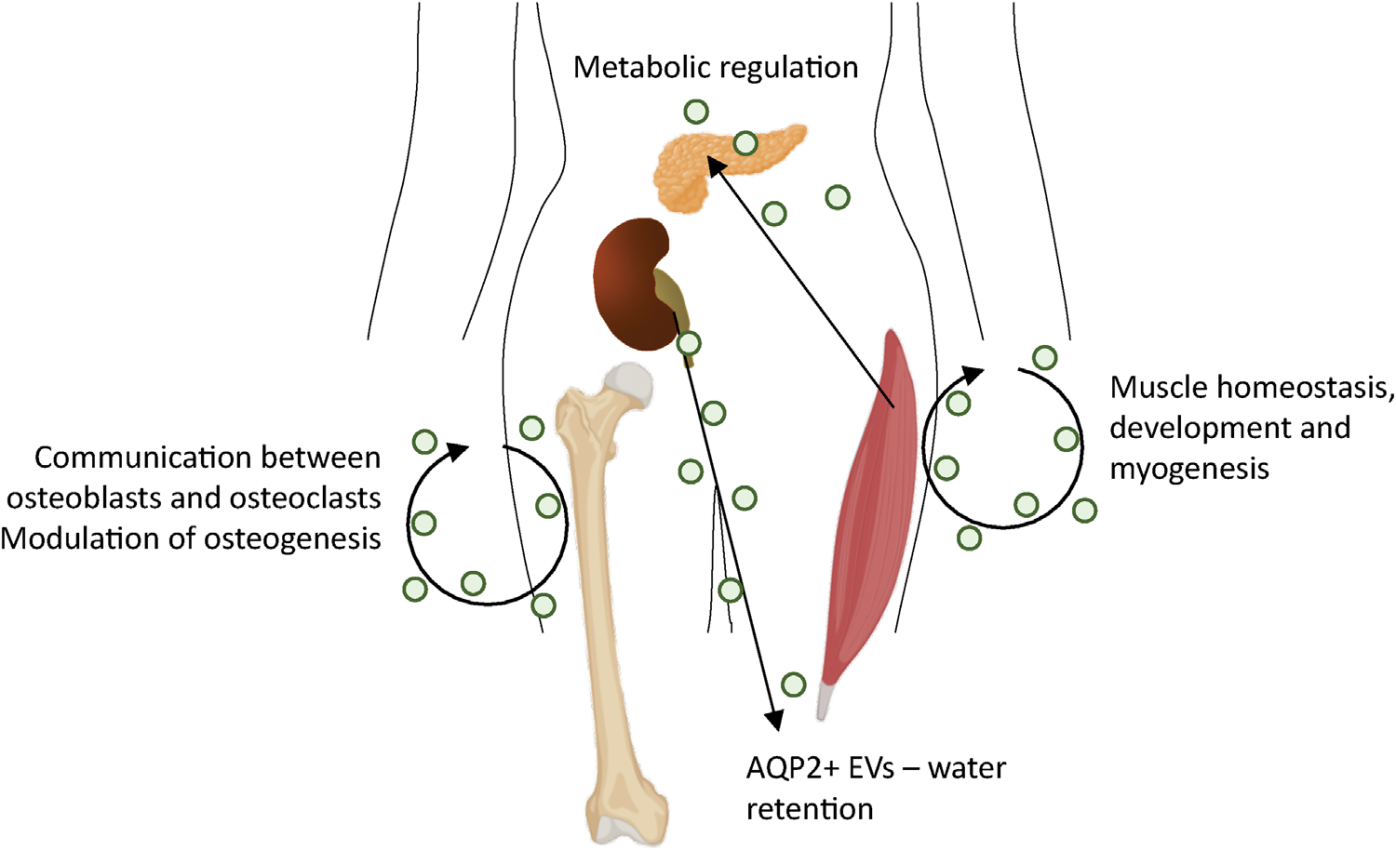
Examples of EV signalling in homeostatic physiological processes. The majority of EV research is based on differences between diseases states and healthy controls, therefore knowledge of how EVs function in maintaining homeostasis is often unclear and understudied in vivo. This figure illustrates our knowledge of some of the key homeostatic processes in the body and the evidence we have of EVs playing a role in vivo. Taken from refs: (Choi et al., 2016; Cobelli et al., 2017; Coenen‐Stass et al., 2016; Davis et al., 2017; Deng et al., 2015; Forterre et al., 2014; Fry et al., 2017; Guescini et al., 2015; Huynh et al., 2016; Jalabert et al., 2016; Le Bihan et al., 2012; Li et al., 2016; Matsuzaka et al., 2016; Miyazawa et al., 2018; Oosthuyzen et al., 2016; Romancino et al., 2013; Street et al., 2011; Sun et al., 2016; Weilner et al., 2016; Whitham et al., 2018)

The musculoskeletal system provides the framework for the body and comprises some of the largest organs by mass. The complexity of regenerative tissue such as bone and muscle, means that intercellular communication is vital in maintaining healthy tissue, as well as effecting repair after damage or during pathology (Figure 3). Much like many of the organ systems, studying muscle or bone‐derived EVs requires the assumption that they are released into the circulation, and therefore necessitates study in a whole animal or a human subject.

### 9.1 Skeletal muscle

The skeletal muscle is the organ of greatest weight in the body, formed by multinucleated myofibers and satellite cells enclosed in basement membrane. The main function of the skeletal muscle is locomotion, but it also contributes to homeostasis and energy metabolism (Henningsen et al., 2010; Pedersen & Febbraio, 2012). Skeletal muscle has been suggested as a significant source of EVs in the blood (Whitham et al., 2018). EV numbers, their cargo and functionality can change under different physiological and pathological muscle conditions (Cobelli et al., 2017) and it has been shown that EVs secreted by skeletal muscle carry different myokines, proteins, miRNAs and mRNA that may play various roles in muscle homeostasis, development and myogenesis (Choi et al., 2016; Forterre et al., 2014; Le Bihan et al., 2012; Romancino et al., 2013). As part of these studies, the muscle ‘secretome’ was analysed, including large and small EVs, enriching proteins from common protein regulation pathways and showing muscle MVs to be taken up other muscle cells. Whilst this secretory capacity has been well established, the biological effect remains unclear and highlights the need for in vivo research.

As in other organ systems, EV cargo plays a key role in the musculoskeletal system. miRNAs such as miR‐1, miR‐133 and miR‐206 are known to be highly enriched in skeletal muscle (Annibalini et al., 2019; Chen et al., 2006). The presence and communication of muscle specific miRNAs in EVs, part of the circulating miRNAs known as ‘myomiRs’, are thought to play a role in controlling muscle homeostasis, proliferation and differentiation (Coenen‐Stass et al., 2016). One of the most abundant miRNA in muscle‐derived EVs is miR‐206, which induces the development and differentiation of tissue, especially after muscle injury during exercise (Guescini et al., 2015). Systemic experiments are starting to show muscle EVs having a much wider effect on different tissues. For example, muscle EVs from mice fed a high‐fat diet seem to be taken up in vivo into the pancreas and, at least using in vitro experiments, can modulate beta cell proliferation, suggesting metabolic regulation mediated by the muscle EVs (Jalabert et al., 2016).

Muscle degeneration in the short term, for example after exercise, affects repair processes. The effects of EVs in short term musculoskeletal repair and regeneration have been widely studied (Cobelli et al., 2017). Muscle damage and calcium increases have been shown to stimulate the release of EV‐associated myomiRs from muscle (Matsuzaka et al., 2016). For example, Choi et al. (Choi et al., 2016) show EVs derived from differentiating myotubes of human skeletal myoblasts can enhance muscle regeneration of myofibers in injury sites, suggested to be through contents of EVs such as insulin‐like growth factor, hepatocyte growth factor, fibroblast growth factor 2 and platelet derived growth factor‐AA. More detailed studies have suggested miR‐206 in the EVs of myogenic progenitor cells may be regulating the expression of muscle extracellular matrix collagen, facilitating fibre growth for muscle repair (Fry et al., 2017).

### 9.2 Bone

The bone is a rich and complex tissue, which undergoes constant communication and remodelling. The hormonal communication between the ‘bone‐making’ osteoblasts and the ‘bone‐recycling’ osteoclasts is commonly documented (Kim et al., 2020), but interest is growing in the role of EVs in these processes, as well as their role in the localised production of synovial fluid. One of the ground‐breaking papers in the EV field was by Valadi et al. (Valadi et al., 2007), who showed that EVs from primary bone marrow derived mouse mast cells contained both mRNA and miRNA, with the mRNA capable of driving protein production in recipient human mast cells. Deng et al. (Deng et al., 2015) later went on to show EVs to communicate from osteoblasts to osteoclasts, transporting receptor activator of nuclear factor kappa beta (RANKL) protein to osteoclast precursors to facilitate their formation. Huynh and colleagues (Huynh et al., 2016) have also demonstrated that mouse marrow osteoclast EVs are enriched in RANKL and inhibit 1,25‐dihydroxyvitamin D3 induced formation, regulating the formation of new osteoclasts. However, as with other fields, this is underexplored in the context of whole physiology.

Bone deterioration is thought to be a normal aging process, and studies of EVs in young vs old volunteers has begun to shed some light on their role in the crosstalk in this microenvironment. Plasma EVs of aging volunteers (over 55) were shown to suppress osteogenesis compared to those of younger participants (younger than 25) (Weilner et al., 2016). The authors believe this action to be through a fall in the concentration of Galectin‐3 in the aging EVs. Blood EVs from elderly volunteers has been shown to have increased levels of osteoclastic miR‐214‐3p, and that this reduced bone formation in mice (Li et al., 2016). Indeed, in osteoclast specific miR‐214‐3p‐transgenic mice, EVs were found to mediate communication between osteoclasts and osteoblasts, suppressing differentiation via inhibition of *Atf4, Alp, Bglap* and *Col1α1* (Sun et al., 2016). Davis and colleagues (Davis et al., 2017) isolated EVs from the bone interstitial fluid of young and aged mice to show that the miR‐183 cluster (miR‐96/182/183) is significantly higher in the EVs from aged animals, and that these EVs inhibit osteogenic differentiation of young bone marrow stem cells. Together these data suggest that circulating EVs change during the aging process, and that changes in cargo and surface proteins may result in functional changes within the skeletal system. However, the incidence of comorbidities with the aging process also encourages the use of whole animal and patient systems to study these processes, in order to understand the intricacies of EV mediated signalling in the bone during normal aging.

## 10 THE GUT MICROBIOME

Microorganisms that inhabit the human body have a critical role in diverse aspects of homeostasis including the modulation of the immune response, production of vitamins, avoidance of colonization by harmful bacteria, to name a few (Integrative, 2019). EVs released by the human microbiota – here called Outer Membrane Vesicles (OMVs) – may play a significant role in health and disease, acting locally and systemically as intercellular communicators to facilitate the aforementioned processes (Nagakubo et al., 2019).

The largest part of the human microbiota inhabits the gastrointestinal system, where they modulate aspects as diverse as insulin signalling, behaviour and allergy (Bunyavanich et al., 2016; Caricilli et al., 2011; Peirce & Alviña, 2019). Recent work has demonstrated that components of the microbiota release OMVs that appear to impact tissue homeostasis and physiological alterations during disease, for example in promoting immune tolerance or activation, and in trafficking of bacterial toxins (Ñahui Palomino et al., 2021). Certainly, the gut balance of the gut microbiota, and the influence of diet and lifestyle on the health of this micro‐organ system, relies heavily on intercommunication between both the microorganisms themselves, as well as the microorganisms and the host. OMVs from the majority of bacteria are between 10–300 nm, and contain similar membrane components to their parent cells – lipids, proteins, lipopolysaccharide, etc. (Kulp & Kuehn, 2010). This makes them broadly slightly smaller than traditional eukaryotic EVs, but nevertheless physicochemically similar. The exact role of OMVs in physiology is currently unclear. However, there is likely to be significant signalling between the microorganism and the host.

To maintain gut homeostasis, microbiota must be in healthy competition with each other, and potentially pathogenic components such as lipopolysaccharide (LPS) must be neutralized by the host. If this maintenance is carried out effectively, this allows the host to maintain appropriate energy levels, lipid homeostasis, inflammatory balance and an effective gut‐blood barrier (GBB). Many bacteria, such as *A.muciphila* (Am) thrive in the healthy gut, and become reduced in inflammatory diseases such as inflammatory bowel disease (Kang et al., 2013). Indeed, the reduced abundance of this bacteria in ulcerative colitis has a detrimental effect on disease progression, and has been correlated with higher inflammatory scores (Earley et al., 2019). Kang and colleagues used EVs derived from Am to decrease the permeability of the GBB during colitis (Kang et al., 2013). As such, OMVs may exert the beneficial effects of the gut microbiota. The presence of toll‐like receptors and other key pathogen‐associated molecular patterns on the surface of many bacterial EVs (Kim et al., 2015; Lee et al., 2015) most likely aids them in their interactions with the host immune system.

## 11 CONCLUSION

Whilst there remain many unknowns about the role of EVs in normal physiological processes, our blossoming understanding of their role in pathology will not only enable the field to expand but also increase our understanding of their function in the healthy body. For more information on the role of EVs in pathology in vivo please see Part II of this series.

## CONFLICT OF INTEREST

Ryan Pink is currently CEO of MetaGuideX, an exosome diagnostics company.
